# Clinical features and magnetic resonance image analysis of 15 cases of demyelinating leukoencephalopathy induced by levamisole

**DOI:** 10.3892/etm.2013.1077

**Published:** 2013-04-24

**Authors:** RUIFANG YAN, QINGWU WU, JIPENG REN, HONGKAI CUI, KAIHUA ZHAI, ZHANSHENG ZHAI, QINGHONG DUAN

**Affiliations:** 1 Center of Medical Imaging, The First Affiliated Hospital of Xinxiang Medical University, Weihui, Henan 453100;; 2 Department of Neurology, The First Affiliated Hospital of Xinxiang Medical University, Weihui, Henan 453100;; 3 Department of Imaging, The Affiliated Hospital of Guiyang Medical University, Guiyang, Guizhou 550004, P.R. China

**Keywords:** levamisole, demyelinating leukoencephalopathy, magnetic resonance imaging

## Abstract

The aim of this study was to explore the diagnostic value of magnetic resonance imaging (MRI) for levamisole-induced demyelinating leukoencephalopathy. The clinical features and MRI findings of 15 patients with levamisole-induced demyelinating leukoencephalopathy were retrospectively analyzed. The abnormality rate of the patients was demonstrated to be 100% by MRI, and scattered multiple cerebral foci were observed in all of the patients. The majority of the foci were located at the centrum ovale, peri-lateral cerebral ventricles and basal ganglia, while the remainder were located in the brain stem and cerebellum, as well as in the white matter regions of the temporal, frontal, apical and occipital lobes. In addition, mottling and ring-shaped enhancements were observed. The study demonstrated that MRI effectively displays demyelinating leukoencephalopathy, and that the combination of MRI with the medical history of the patient is of significance for the early diagnosis, differentiation and treatment of demyelinating leukoencephalopathy.

## Introduction

Levamisole is a broad spectrum anthelmintic agent which is primarily used for expelling *Ascaris* and hookworms. In addition, it enhances the functions of phagocytes and T cells, and strengthens immune function (1,2,3). As a result, levamisole is also used as an immunoregulant for certain immune-related diseases, in order to increase the resistance of patients to bacterial and viral infections. Levamisole may be applied in the treatment of nephritic syndrome (4), dermatological diseases (5), recurrent aphthous ulcers (6) and inflammatory bowel disease (7). In addition, levamisole, in combination with 5-fluorouracil (5-FU), reduces the clinical recurrence and overall mortality rates among patients with stage III colon cancer by 41 and 33%, respectively (8). However, in 1992, Hook *et al* (9) revealed that the combination of 5-FU and levamisole may lead to serious multifocal leukoencephalopathy. Since then, cases of demyelinating leukoencephalopathy induced by an overdose of levamisole have become increasingly common (10–12). In China, levamisole has been widely applied as an insect repellent, despite the fact that a normal dose is sufficient to induce demyelinating leukoencephalopathy in certain patients (13,14). The awareness that a single administration of levamisole, or its combined use with 5-FU, may induce demyelinating leukoencephalopathy has increased; however, diagnostic errors, missed diagnoses and delays in treatment frequently occur in clinic, due to the medical history of the patients being overlooked, in addition to the long-term latency of the disease and various other disease factors. As a consequence, a number of sequelae may develop. Magnetic resonance imaging (MRI) is an important method of examination for demyelinating leukoencephalopathy. However, while MRI effectively displays brain lesions, the differentiation of demyelinating leukoencephalopathy from other diseases remains challenging.

The current study analyzed the MRI data of 15 patients with levamisole-induced demyelinating leukoencephalopathy, who received treatment at the First Affiliated Hospital of Xinxiang Medical University (Weihui, China) between January 2002 and June 2011. The aim of the investigation was to further enhance the awareness of demyelinating leukoencephalopathy, to reduce the sequelae caused by diagnostic errors and missed diagnoses and to increase the diagnostic level of MRI for this disease.

## Patients and methods

### Patient data

A total of 15 patients with demyelinating leukoencephalopathy were accepted for this study. Of the patients, five were males and 10 were females, and the age range was between 31 and 54 years (mean age, 45.5±8.67 years). All patients had a history of levamisole use for worm expulsion, between two weeks and two months prior to the disease onset, with a dose range of 50–150 mg. According to the typical clinical and diagnostic features of imidazole-induced delayed cerebral diseases (15–18), and the seven diagnostic criteria proposed by Zheng in 1995 (19), a history of anthelmintic contact prior to the occurrence of the disease is the first condition for disease diagnosis. All patients in the study met this criterion; in addition, at least two types of ancillary examination supported the diagnosis, such as cerebrospinal fluid (CSF) examination, electroencephalography (EEG) and head MRI.

This study was conducted in accordance with the Declaration of Helsinki, and approved by the ethics committee of The First Affiliated Hospital of Xinxiang Medical University. Written informed consent was obtained from all participants.

### Clinical data

Of the 15 patients, three presented with a fever, eight had limb weakness (accompanied by dizziness and a headache in two cases, and a fever in three cases), one had apathia/was emotionally disturbed, one had limb myasthenia and progressive language disorders and two had impaired vision. Nine patients underwent a CSF examination, and the results demonstrated that four had mild inflammatory changes. The white blood cell counts that were obtained ranged from 8×10^9^ to 1.2×10^11^/l (predominated by monocytes), but the levels of glucose and chloride were essentially normal. Six patients did not exhibit any abnormalities. The EEG results of the 15 patients revealed high-amplitude and slow waves, which suggested moderate or severe abnormalities. There was an absence of evident waveforms in the bilateral visual evoked potentials of two patients; however, no abnormalities in the upper limb somatosensory evoked potentials were observed. One patient presented with negative P14, N20, P28, and N35 waveforms (indicative of a central impairment), and a prolonged latent period of P100 with a low wave amplitude in visual evoked potentials.

### MRI

All patients were subjected to MRI [including spin echo T1-weighted imaging (SE T1WI), T2 fluid-attenuated inversion recovery (FLAIR), heavy T1-weighted imaging (T2WI) and diffusion-weighted imaging (DWI)], using the Signa 1.5T MRI system (GE Healthcare, Waukesha, WI, USA). The scanning parameters were as follows: SE T1WI: TR: 620 msec, TE: 12 msec, FOV: 24×24 cm, matrix: 200×256, thk5mm/sp1.5mm, NEX: 2. T2 FLAIR: TR: 8,000 msec, TE: 136 msec, TI: 2,000 msec, FOV: 24×24 cm, matrix: 512×512, thk5mm/sp1.5mm, NEX: 2. DWI/T2WI: SE/EPI, TR: 3,600 msec, TE: 70.3 msec, FOV: 24×24 cm, matrix: 256×256, thk5mm/sp1.5mm, NEX: 2. In addition, one patient underwent a contrast-enhanced scan, using gadopentetate dimeglumine as a contrast medium.

## Results

### MRI

**Figure 1.**
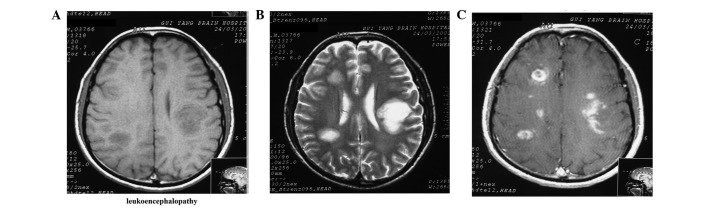
Case 1: (A) Axial T1-weighted imaging (T1WI) exhibits hypointense multiple foci at the bilateral peri-lateral ventricles and centrum ovale; (B) axial heavy T1-weighted imaging (T2WI) exhibits hyperintense foci; and (C) axial enhancement demonstrates patch-, ring- and ring cleavage-like foci.

**Figure 2.**
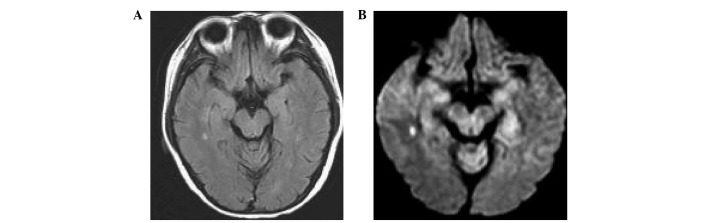
Case 2: (A) Axial T2 fluid-attenuated inversion recovery (FLAIR) exhibits spot-like hyperintensity, and (B) axial diffusion-weighted imaging (DWI) exhibits hyperintense foci at the right temporal lobe.

**Figure 3.**
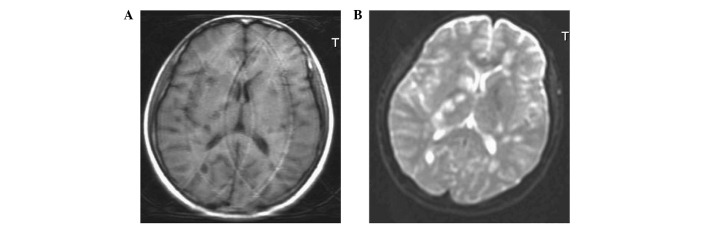
Case 3: (A) Axial T1-weighted imaging (T1WI) demonstrates multiple hypointense foci at the bilateral basal nuclei; and (B) diffusion-weighted imaging (DWI) exhibits hyperintense foci.

The MRI results demonstrated a 100% abnormality rate in the patients. The majority of the foci were located at the bilateral centrum ovale, peri-lateral ventricles and basal ganglia, while the remaining foci were observed in the white matter areas of the frontal, apical, occipital, and temporal lobes, as well as in the brain stem and cerebellum (Figs. 1–3). All patients (100%) were revealed to have foci present at the bilateral centrum ovale and peri-lateral ventricles, while 10 patients (66.7%) exhibited foci at the basal ganglia, four (26.7%) at the temporal lobe, one (6.7%) at the frontal lobe, one (6.7%) at the apical lobe, one (6.7%) at the occipital lobe and one (6.7%) in the brain stem and cerebellum.

The foci observed in the MRI results were of different sizes, with large foci present in round sheets, and small foci appearing patchy, with indistinct boundaries (Figs. 1–3). Irregular patchy foci were observed in six of the patients (40%), whereas the predominant round and mass-like foci were observed in all patients (100%).

The foci were observed to be hypointense by T1WI, hyperintense with clear boundaries by T2 FLAIR, hyperintense by T2WI and predominantly hyperintense by DWI (Figs. 1–3). Three patients displayed mild edema surrounding the foci.

One patient was demonstrated to have enhanced demyelinating leukoencephalopathy, and axial enhancement revealed the majority of the foci to be patch-, ring- and arc-like (Fig. 1C).

### Treatment effectiveness

Hormone therapy was observed to be effective for all the patients. The patients were discharged from the hospital following improvement or recovery.

## Discussion

Levamisole-induced cerebral disease is classified as a delayed demyelinating leukoencephalopathy. The disease exhibits many pathological similarities to acute disseminated encephalomyelitis (ADEM) and multiple sclerosis (MS) (20). Demyelinating leukoencephalopathy is characterized by a number of clinical features, and occurs within two months of levamisole administration. The majority of cases have an acute or subacute onset, which manifests as diffuse brain injury, early mental symptoms and movement disorders. CSF examinations of the patients appear normal, or with a marginally raised white blood cell count, while EEG and MRI examinations demonstrate diffuse slow waves and cerebral white matter demyelination, respectively. Short-term cortical hormonal therapy may achieve a successful outcome (21). In the present study, eight patients presented with movement disorders as the primary symptom, and two presented with headaches, dizziness or emotional disturbances. Based on these symptoms, it was possible to exclude a diagnosis of brain parenchymal disease by computed tomography or MRI. Two patients initially presented with impaired vision, and no obvious waveforms were observed in their visual evoked potentials. It was suggested that one of the patients had a central impairment. Based on these symptoms, it was not possible to exclude the possibility that the visual impairment was caused by a cerebral disease. It is necessary to enhance the awareness of the significance of ancillary examinations, in order to increase the diagnostic rate of demyelinating leukoencephalopathy.

Clinically, levamisole-induced demyelinating leukoencephalopathy improves or recovers following treatment; however, its radiological manifestations, with regard to lesion distribution, morphology, signals, enhancement effects and dynamic changes, have been demonstrated to appear later than the clinical signs of the disease (10,20,22). Although MRI provides an important diagnostic basis for demyelinating leukoencephalopathy, the differentiation of the disease from ADEM and MS remains challenging. For multifocal leukoencephalopathy, demyelinating lesions may be easily diagnosed by MRI. However, it is still necessary to differentiate demylelinating leukoencephalopathy from other diseases, as follows. With regard to MS, differentiation may be achieved due to the fact that the majority of the cases of demyelinating leukoencephalopathy present with fever in the clinic, and the MRI results exhibit foci that are larger than those of MS. In addition, the foci in demyelinating leukoencephalopathy appear in round, as opposed to rectangular, sheets. By contrast, the differentiation between demyelinating leukoencephalopathy and ADEM is predominantly based on the clinical symptoms of the disease and the outcome of the CSF examination. However, in certain cases the diagnosis remains difficult. In such cases, a repeated inquiry into the case history is required. In addition to MS and ADEM, it may be necessary to differentiate between demyelinating leukoencephalopathy and metastatic tumors, since diffuse mass-like foci may be observed in certain cases of demyelinating leukoencephalopathy. This differentiation may be achieved through the observation of peripheral edema, with an apparent space -occupying effect. Peripheral edema is not observed in demyelinating leukoencephalopathy. Furthermore, metastatic tumors exhibit characteristic enhancement features, where the enhancements appear node-like or uneven ring-shaped. By contrast, enhancements may be absent in demyelinating leukoencephalopathy, or the enhancements may appear stippled or arc-like.

Although the MRI manifestations of demyelinating leukoencephalopathy are not specific, the outcomes of the MRI, in combination with the focal morphology, location and enhancement phenomena, may indicate a potential diagnosis. The diagnosis of demyelinating leukoencephalopathy may then be confirmed if the patient has a recent history of insect repellent use.

In conclusion, MRI effectively demonstrates cerebral white matter demyelination. The appearance of multiple cerebral demyelination lesions is indicative that demyelinating leukoencephalopathy may be considered as a potential diagnosis. Based on this consideration, it is necessary to establish the medical history of the patient, prior to the disease occurrence. Once the diagnosis of demyelinating leukoencephalopathy is confirmed, a clinical treatment protocol may be devised.
